# Fibroblast growth factor 21 (FGF21) is increased in MDD and interacts with body mass index (BMI) to affect depression trajectory

**DOI:** 10.1038/s41398-021-01679-y

**Published:** 2022-01-11

**Authors:** Brittany L. Mason, Abu Minhajuddin, Andrew H. Czysz, Manish K. Jha, Bharathi S. Gadad, Taryn L. Mayes, Madhukar H. Trivedi

**Affiliations:** 1 Department of Psychiatry, UT Southwestern Medical Center, Dallas, TX USA; 2 Department of Population and Data Sciences, UT Southwestern Medical Center, Dallas, TX USA; 3 Department of Psychiatry, Texas Tech University Health Sciences Center El Paso, Dallas, TX USA

**Keywords:** Predictive markers, Psychology

## Abstract

Fibroblast growth factor 21 (FGF21) is a key regulator of metabolic function and nutrient preference. It also affects biological pathways associated with major depressive disorder (MDD), including corticotrophin-releasing hormone (CRH), leptin, and sympathetic activity. Lower levels of cerebrospinal fluid FGF21 have been associated with higher Beck Depression Inventory scores. FGF21 was examined as a metabolic marker that could be associated with MDD and evaluated as a biomarker of antidepressant treatment response in a large, randomized placebo-controlled trial in chronic, early-onset MDD participants. FGF21 levels at baseline and during treatment were determined for participants in the Establishing Moderators and Biosignatures of Antidepressant Response for Clinical Care (EMBARC) study. FGF21 was analyzed by ELISA in individuals with chronic, early-onset MDD (first major depressive episode before 30 years) compared to healthy control participants. Participants with MDD had higher levels of FGF21 compared to healthy controls (HCs), even after controlling for baseline age, sex, race, Hispanic ethnicity, BMI, and site (*β*-coefficient = 1.20, *p* < 0.0001, Cohen’s *d* = 0.60*)*. FGF21 did not change over time nor differ between treatment groups. Interestingly though, those with normal BMI and lower FGF21 levels showed a reduction in depression severity over time compared to all other groups. In conclusion, depression is associated with higher levels of FGF21 compared to healthy controls and those with lower levels of FGF21 (25th percentile of the sample) in the context of normal-weight BMI seem to have improved depression severity over time.

## Introduction

Obesity and associated metabolic dysfunction have been shown to significantly impact the biology, disease course, and treatment response of major depressive disorder (MDD) [1, 2], and yet the basic mechanisms underlying these associations are largely under-recognized. Understanding the specific biological mechanisms related to the development of depression and related outcomes are essential to target and refine treatment selection within the heterogeneity of depression.

Fibroblast growth factor 21 (FGF21) is a key hormonal regulator of metabolic function and nutrient preference. Its production can be induced in the liver by diverse metabolic stressors, including starvation and protein-deficient diets, as well as simple sugars and ethanol, and has been shown to act directly on the nervous system to suppress sweet and ethanol preference, increase thermogenesis, and improve insulin sensitivity [3, 4]. It is known to affect biological signaling cascades implicated in depression, including the corticotrophin-releasing hormone (CRH), leptin, and the sympathetic nervous system pathways [4]. FGF21 is also associated with poor metabolic health, with serum FGF21 levels being positively correlated with adiposity, fasting insulin and triglycerides, and increased risk of metabolic syndrome associated with high FGF21 [5]. This increased risk in the context of higher adiposity may be related to FGF21 resistance, which has been demonstrated in obese mice having significantly reduced FGF21 signaling response in both liver and fat tissues following exogenous administration of FGF21 [6]. FGF21 was not associated with stress-induced increase in cortisol in humans 3 months following a period of chronic stress, but was positively correlated with self-reported ability to cope with the stress [7], suggested that FGF21 may also play a role in managing the consequences of psychological stress. There are fewer data to support how FGF21 may be related to psychiatric conditions that also involve psychological stress, such as MDD.

FGF21 has been previously examined in depression and bipolar disorder in small studies. Lower levels of FGF21 in the cerebrospinal fluid (CSF) was associated with higher scores on the Beck Depression Inventory in Chinese men, but not women, who were not diagnosed with a mood disorder [8]. Changes in FGF21 following valproate treatment for bipolar depression in bipolar II disorder were associated with Hamilton Depression Rating Scale (HDRS) scores, with individuals having increased FGF21 showing worse outcomes as measured by HDRS scores [9]. Given these associations, FGF21 was explored as a metabolic marker that could be associated with MDD and evaluated as a biomarker of treatment response in a large, randomized, placebo-controlled trial of sertraline in chronic, early-onset MDD participants. The following questions were examined:

1) Are there differences in FGF21 levels in those with MDD compared to Healthy Controls?

2) Among participants with MDD, do levels of FGF21 change over 8 weeks of treatment, and are there demographic, clinical, or treatment characteristics that influence changes in FGF21 over time?

3) Are changes in depression severity associated with baseline FGF21 in the context of body mass index (BMI)?

## Participants and methods

### Participants

The Establishing Moderators and Biosignatures of Antidepressant Response for Clinical Care (EMBARC) study was a randomized, double-blind, placebo-controlled trial of the serotonin selective reuptake inhibitor sertraline. Full rationale and design has been detailed previously [10]. The study enrolled 296 participants across four sites: the University of Texas Southwestern Medical Center in Dallas, TX; Columbia University in New York, NY; the University of Michigan in Ann Arbor, MI; and Massachusetts General Hospital in Boston, MA. Enrolled MDD participants were 18–65 years old, fluent in English, and had chronic, early-onset MDD (first major depressive episode prior to age 30 years) as diagnosed by the Structural Clinical Interview for DSM-IV. MDD participants were excluded if they scored <14 on the Quick Inventory of Depressive Symptomatology–Self Report baseline, had any other primary mental disorder other than MDD, had a lifetime history of a psychotic or bipolar disorder, had a substance abuse disorder within 6 months (except for nicotine dependence), or had the presence of a medical condition that would compromise MDD-specific findings. Forty healthy control participants were also enrolled, who had no personal history of or first-degree relative with a mood disorder.

This study was approved by the Institutional Review Board at each institution and all participants signed informed consent. Enrollment was active from 2011 to 2015. The study was registered with ClincialTrials.gov (NCT01407094).

### Treatment and assessments

Upon meeting all inclusion and no exclusion criteria, MDD participants were randomized to double-blind, 8-week course of either sertraline or placebo in stage 1 of the study. Randomization was in 1 : 1 ratio and stratified by site. Dosing, which was started at 50 mg of sertraline or similar placebo, with potential weekly titration of 50 mg/day up to 200 mg/day of sertraline or similarly increased placebo, was based on clinical decision, guided by measurement-based care principles. At the end of stage 1, responders continued their respective active treatment or placebo. Non-responders in the sertraline arm were switched to bupropion, whereas those in the placebo arm were switched to sertraline. Both MDD and healthy control subjects were followed for 16 weeks. Only data from stage 1 of the study were analyzed for this report.

A range of clinical characteristics were collected during the EMBARC study, including symptoms of depression, mania, anxiety, anger, and suicide, as well as physical and medical characteristics, such as pain, sexual functioning, adverse effects, etc. (see [10] for full details). Outcomes on severity of depressive symptoms and changes over time were evaluated using HDRS (HAMD-17 [11]), which was collected at Baseline and at post-randomization Weeks 1, 2, 3, 4, 6, and 8 during the stage 1 of the study.

### Body mass index

Participant’s vital signs were obtained at each visit and BMI was calculated at each time point. In general, BMI was evaluated as a continuous measure. For categorical displays of BMI, standard BMI categories of normal weight (18.5–24.9 kg/m^2^), overweight (25–29.9 kg/m^2^), obese (30–34.9 kg/m^2^), and morbidly obese (>35 kg/m^2^) were used.

### Blood collection and processing

Venous blood samples were collected in EDTA tubes at Baseline for all participants and at Weeks 1 and 8 for MDD participants. The samples were then isolated for plasma at each site. Plasma aliquots were frozen and stored at −80 °C until frozen shipment to the Rutgers University Cell & DNA Repository (RUCDR). The full set of plasma samples from all sites was received frozen from RUDCR and stored at −80 °C until analysis.

### Quantification of analyte

FGF21 was analyzed using analyte-specific enzyme-linked immunoabsorbant assays (FGF21: BioVendor Czech Republic), with a required dilution step to ensure that values would fall within the limits of detection and were adjusted for dilution after assay as is the standard practice. The limit of detection was 7 pg/mL and the inter-assay variability was *n* = 6; coefficient of variation (CV) = 3.3%. Interplate controls were used to detect any batch effects. In general, FGF21 was evaluated as a continuous measure. Low and high categories of FGF21 were defined as the 25th percentile (low) and the 75th percentile (high) of the sample.

### Statistical analyses

FGF21 concentrations were calculated as described and adjusted for the dilution factor. The control values were evaluated for batch effects by scatterplot and no batch effect was detected for this sample set. All values were log_2_-transformed for use in statistical comparisons. Some samples measured were below the lower limit of detection and could not be included in analyses.

Continuous data were summarized as mean ± SD or median and interquartile range. Categorical data were summarized as frequency and percentages.

Analyses of covariance (ANCOVA) was used to assess whether baseline level of log_2_-transformed FGF21 were different between the MDD and HC control samples. Baseline age, sex, race, Hispanic ethnicity, BMI, and study site were used as covariates. In the MDD sample, baseline levels of log_2_-transformed FGF21 were compared between sertraline and placebo groups using ANCOVA analyses with baseline levels of depression severity (as measured by HAMD-17), baseline age, sex, race, Hispanic ethnicity, BMI, and study sites as covariates.

To assess whether FGF21 levels changed during treatment in the MDD sample, a repeated-measures ANCOVA (repeated ANCOVA) analysis was used with log_2_-transformed FGF21 levels as outcome, time (Week 0, Week 1, and Week 8) as a within-subject factor, and randomization groups (sertraline vs. placebo) as a between-subject factor and the interaction between them. Baseline depression (HAMD-17) severity, baseline age, sex, race, Hispanic ethnicity, BMI, and study site were included in the model as covariates. If the Group × Time interaction effect was statistically significant, the two treatment groups were compared at Weeks 0, 1, and 8.

To assess the effects of baseline FGF21 levels on the changes in depression severity in the MDD sample, a repeated ANCOVA analysis was used with HAMD-17 at Baseline and Weeks 1, 2, 3, 4, 6, and 8 as the outcome, time as the within-subject factor, and baseline FGF21 levels as the covariate of interest. Time by FGF21 interaction was also included in the model. Given the known differences of FGF21 levels across obesity levels [12], participant BMI, time by BMI interaction, as well as a three-way interaction between time, FGF21, and BMI were also included in the model. In addition, baseline age, sex, race, Hispanic ethnicity, treatment group, and study site were included in the ANCOVA model.

All analyses were done using SAS 9.4 (SAS, Inc., Cary, NC) and statistical significance was assessed at *p* < 0.05. At this time, no adjustments were made for multiple comparison.

## Results

**Fig. 1:**
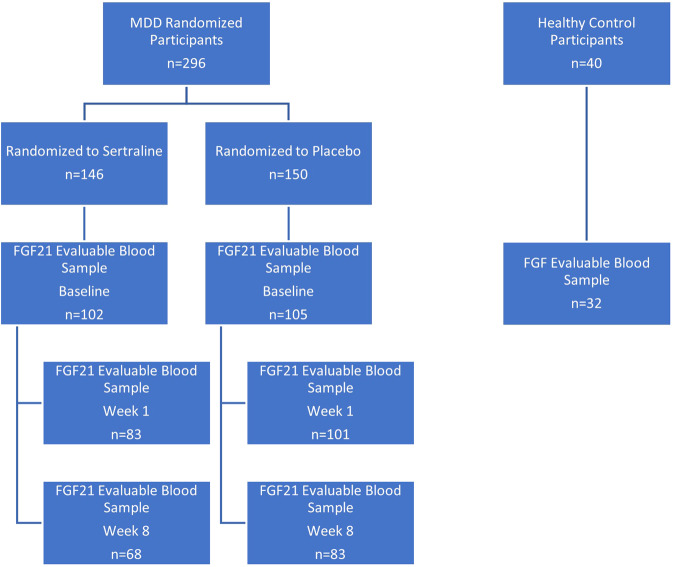
Consort diagram.

EMBARC enrolled 309 participants with MDD and 40 healthy controls across the four sites. Of these 309 participants with MDD, the first 10 participants with MDD were randomized to citalopram and 3 participants were ineligibly randomized. Thus, the modified intent-to-treat stage 1 EMBARC sample included 296 participants randomized to sertraline or placebo. Not all participants provided a blood sample at every blood collection visit and not all participants were retained through all of stage 1. Of these, 207 provided blood samples that were able to be evaluated for FGF21 (102 sertraline and 105 placebo). Among the 40 HC, 32 provided blood samples that were able to be evaluated FGF21. Thus, the modified intent-to-treat sample for this report includes 207 participants with MDD, who were randomized and 32 HC participants. Among the MDD sample, 184 provided blood samples that were able to be evaluated FGF21 at Week 1 (83 sertraline and 101 placebo) and 151 provided blood samples that were able to be evaluated for FGF21 at Week 8 (68 sertraline and 83 placebo) (Fig. 1). Of the samples analyzed for FGF21, 32 of these samples were below the lower limit of detection and not included in the analyses.

The demographic and clinical characteristics of the sample are presented in Table 1. The majority of participants were female (67.6% MDD and 62.5% HC), with a mean age around 38 years. Most were non-Hispanic and Caucasian. MDD participants had higher BMI (*p* = 0.029) and HAMD-17 scores (*p* < 0.0001). There were no other significant differences between the MDD and HC samples, and there were no statistical differences between the sertraline and placebo participants.

**Table 1:** Sample demographics.

Variable	MDD *n* = 207	HC *n* = 32	*p*-Value
Age, years, mean (SD)	38.9 (13.2)	38.8 (15.1)	0.988
Sex, *n* (%)			0.552
Female	140 (67.6)	20 (62.5)	
Male	67 (32.4)	12 (37.5)	
Race, *n* (%)			0.710
White	138 (66.7)	30 (67.5)	
African American	39 (18.8)	8 (25.0)	
Other	30 (14.5)	4 (12.5)	
Ethnicity			0.082
Hispanic	40 (19.3)	2 (6.2)	
Non-Hispanic	167 (80.7)	30 (93.8)	
Site			0.005
Columbia	63 (30.4)	9 (28.1)	
Massachusetts General	16 (7.7)	9 (28.1)	
Michigan	51 (24.6)	7 (21.9)	
UTSW	77 (37.2)	7 (21.9)	
BMI, mean (SD), kg	29.1 (7.5)	25.7 (5.8)	0.029
HAMD-17, mean (SD)	18.5 (4.5)	0.6 (0.8)	<0.0001
Log_2_ (FGF21)	7.3 (1.3)	6.3 (1.5)	<0.0001

BMI values missing for 8 HC and 22 MDD patients.

### Are there differences in FGF21 levels in those with MDD compared to Healthy Controls?

Mean FGF21 level for MDD participants at Baseline was significantly higher compared to that for HC participants (7.3 ± 1.3 pg/mL vs. 6.3 ± 1.5 pg/mL; *p* < 0.0001), even after adjusting for baseline age, sex, race, Hispanic ethnicity, BMI, and site (*β*-coefficient = 1.20, *p* < 0.0001, Cohen’s *d* = 0.60).

### Among participants with MDD, do levels of FGF21 change over 8 weeks of treatment, and are there demographic, clinical, or treatment characteristics that influence changes in FGF21 over time?

Table 2 provides the mean FGF21 levels at Baseline and Weeks 1 and 8 for MDD participants. Repeated ANCOVA (adjusted for baseline HAMD-17, baseline age, sex, race Hispanic ethnicity, BMI, and study site) revealed no changes in FGF21 levels over the 8-week treatment phase (main effect of time *p* = 0.4411) or between participants in sertraline and placebo groups (main effect of treatment group *p*-value = 0.7978). In addition, there was no significant time by treatment group interaction (*p* = 0.6116), suggesting that participants in the two treatment groups did not show differences in FGF21 changes over the 8 weeks. Other factors examined as covariates also did not impact change in FGF21 over 8 weeks, with the exception of baseline age, with older age being associated with greater change in FGF21 level (Table 3).

**Table 2:** Log_2_ FGF21 levels over the course of treatment for sertraline vs. placebo.

	Sertraline	Placebo	Test statistic (*p*-value)
Baseline	7.3 (1.3)	7.4 (1.2)	0.9 (0.390)
Week 1	7.1 (1.5)	7.2 (1.2)	0.1 (0.705)
Week 8	7.2 (1.5)	7.2 (1.4)	0.2 (0.788)

**Table 3:** Change in FGF21 levels over 8 weeks by treatment group by Stage 1 treatment.

Source	Numerator DF	Denominator DF	*F*-value	*p*-Value
Time	2	299	0.82	0.4411
Treatment group	1	187	0.07	0.7978
Time × treatment group	2	300	0.49	0.6116
HAMD-17 at Baseline	1	187	0.76	0.3833
Age at evaluation	1	179	12.75	0.0005
Sex	1	187	0.29	0.5880
Race	2	187	2.70	0.0698
Hispanic ethnicity	1	181	1.17	0.2800
Site	3	198	1.11	0.3456
BMI	1	189	3.48	0.0637

### Are changes in depression severity associated with baseline FGF21 in the context of BMI?

**Fig. 2:**
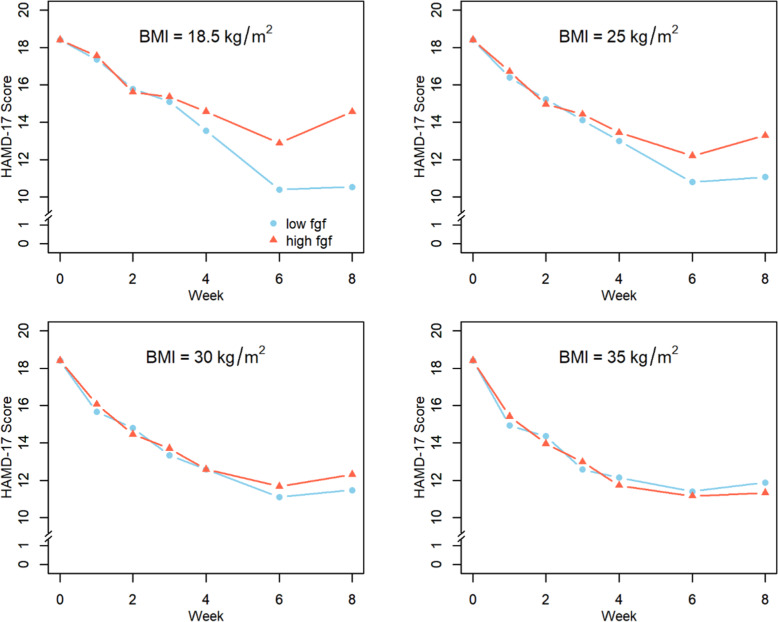
Change in HAMD-17 score over 8 weeks for participants with low versus high FGF21. BMI of 18.5–24.9 kg/m2 (normal weight), 25–29.9 kg/m2 (overweight), 30–34.9 kg/m2 (obese I), and 35 kg/m2 (obese II). Low and high FGF21 are defined as 25th and 75th percentiles, respectively.

A significant three-way interaction for FGF21, time based on repeated ANCOVA, and BMI was observed, suggesting that the effect of baseline FGF21 on changes in HAMD-17 over time depends on the BMI levels of participants (*F* = 3.52, d.f. = 6, 742, *p* = 0.0019). Specifically, among patients with normal BMI, those with low FGF21 (FGF21 = 95.6 pg/mL at baseline, 25th percentile) had significantly greater reduction in HAMD-17 score over time compared to participants with high FGF21 (FGF21 = 293.3 pg/mL at baseline, 75th percentile). However, there were no significant differences in HAMD-17 change over time based on low or high FGF21 in the other groups of participants who were overweight or obese. In addition, main effects of time, baseline HAMD total score, and site, as well as time × BMI and time × baseline FGF21 interactions were also associated with HAMD-17 scores. See Table 4 and Fig. 2 for details.

**Table 4:** Change in HAMD-17 scores over 8 weeks by levels of baseline FGF21 and BMI.

Effect	Num DF	Den DF	*F*-value	*p*-Value
Time	5	762	5.95	<0.0001
BMI	1	166	1.03	0.3105
BMI × Time	5	760	4.52	0.0005
FGF21 at Baseline	1	169	2.28	0.1330
FGF21 × Time	5	763	4.35	0.0007
BMI × FGF21 × Time	6	742	3.52	0.0019
HAMD-17 at Baseline	1	164	36.73	<0.0001
Age	1	160	7.40	0.0072
Sex	1	161	0.61	0.4345
Race	2	163	0.17	0.8413
Hispanic ethnicity	1	162	2.18	0.1417
Site	3	164	5.98	0.0007
Treatment group	1	162	0.67	0.4142

## Discussion

Using data from the EMBARC study, FGF21 was found to be higher in those with MDD compared to HC and FGF21 remained relatively stable over time and was not impacted by depression severity or by treatment. Importantly, when FGF21 levels and BMI were considered together, a significant three-way interaction was found. Participants with lower BMI, classified as normal weight, and lower FGF21 being in the 25th percentile of the sample had a greater decrease in depression severity as measured by the HAMD-17 compared to those with higher FGF21 being in the 75th percentile of the sample. There was no difference for participants with higher BMI, including those classified as overweight or obese, regardless of their levels of FGF21. These findings could indicate that elevation in FGF21 and elevated BMI may be biomarkers of treatment resistance.

The results from this study strengthen the evidence that increased FGF21 is associated with MDD and may have an impact on outcomes for certain individuals (i.e., those of normal weight). The finding that FGF21 was higher in individuals with MDD is somewhat in contrast to previous research showing that lower levels of CSF FGF21 were associated with higher BDI scores in Chinese men, but not women, and without official diagnosis of depression [8]. This finding was from a small sample size and taken from the CSF and not plasma, which may affect this relationship even though FGF21 can cross the blood–brain barrier [13]. Furthermore, all participants in the study by Liu and colleagues had BDI scores less than 17, indicating no more than mild depressive symptoms.

Identification of the interaction between FGF21, BMI, and depression improvement over time suggests that FGF21 is important for people with MDD, who have a lower BMI, but for obese patients other mechanisms may be related to treatment outcomes. FGF21 acts on both peripheral and central targets [4], and has been shown to regulate preference for both sweet and alcohol, which may be dopamine-related [3, 14]. As dopamine signaling is implicated in mood regulation and reward perception [15], this could suggest that some aspect of FGF21 signaling is directly related to mood. However, given the numerous biological systems that are affected by FGF21, including activation of the sympathetic nervous system or indirect action on CRH [16], determining how FGF21 contributes to mood and the development of MDD will require further research. It is unclear whether the results suggest some effect contributing directly to the expression of depressed mood or instead suggest that FGF21 resistance in the system more broadly may be associated with a decreased ability to respond to treatment. The attenuated change in depression severity in those normal-weight participants with higher FGF21 does suggest that some aspect of FGF21 signaling is perpetuating depressive symptoms.

There are several limitations of this report. First, the sample size of healthy controls is relatively small and larger sample sizes are needed to confirm the findings. Second, the inclusion and exclusion criteria for the EMBARC study may limit the generalizability of these findings. Selection of FGF21 as a metabolic biomarker of interest may not be adequate to fully understand the interplay between metabolic biomarkers and depression symptoms, in particular given that weight likely plays an important role in metabolic markers, as evidenced by the results.

Importantly, these analyses highlight the interaction of BMI with the potential of a biomarker effect. The relationship between BMI and increased psychiatric symptoms, including MDD, is well known [17–19]; however, BMI is not frequently included as a variable by which treatment effects are evaluated. Simply controlling for this variability in the model may hide key biological differences in these responses. Given the different physiological states present in those with obesity, it is wise to consider that biological responses or treatment responses likely differ dependent on the metabolic state of the person. These analyses indicated that other factors may influence levels of FGF21 such differences between sexes or between racial and ethnic groups; however, larger studies have indicated that such interactions have not been significant and instead FGF21 was determined to be valuable as a biomarker for metabolic syndrome in multi-ethnic populations [20, 21]. Thus, these findings from the EMBARC study support the association of FGF21 with MDD and provide further evidence that metabolic changes in MDD are worthy of further study as biomarkers. In addition, inclusion of BMI as a specific factor by which groups are stratified may further clarify how biological factors or biomarkers of interest may be differentially impacted. BMI could be a clinically useful marker, as it is easily obtained in clinical practice and could lead to better understanding of treatment response and non-response, particularly in the context of metabolic markers like FGF21.
